# Research on the Effect of Authenticity on Revisit Intention in Heritage Tourism

**DOI:** 10.3389/fpsyg.2022.883380

**Published:** 2022-05-30

**Authors:** Gefen Zhou, Wenkuan Chen, Yuting Wu

**Affiliations:** ^1^Business and Tourism School, Sichuan Agricultural University, Dujiangyan, China; ^2^College of Management, Sichuan Agricultural University, Chengdu, China

**Keywords:** object-based authenticity, existential authenticity, memorable tourism experience (MTE), place attachment, revisit intention

## Abstract

The authenticity of heritage tourism is an important factor for attracting tourists. Research has shown that authenticity is related to revisit intention. However, little attention has been paid to the impact of heritage tourism authenticity on revisit intention. Drawing on cognitive appraisal theory, we constructed a model of the mechanism underlying this relationship. Questionnaires were distributed at one world heritage site (the Dujiangyan irrigation system) in China, and data from 596 valid cases were collected. Using structural equation modeling, the results showed that authenticity, directly and indirectly, affects tourists' revisit intention via memorable tourism experiences and place attachment. The current paper enriches existing literature on the relationship between authenticity and revisit intention and provides a theoretical basis for promoting authenticity and revisit intention in heritage tourism.

## Introduction

As a way for tourism to promote people to experience symbols and projects from different historical periods, heritage tourism has been extensively explored by academics. Authenticity is the core attribute of heritage tourism (Lee et al., 2016) and is regarded as an original and common value (Frisvoll, 2013). It is an important factor for tourists to experience tourist attractions set in different historical periods (Frisvoll, 2013). Indeed, one of the primary aims of heritage tourism is the pursuit of authenticity (Park et al., 2019). However, the current understanding of authenticity has changed; the focus has shifted from object-based to subject-based tourism (Wang, 1999). Therefore, authenticity connects the supply and demand elements of heritage tourism (Lu et al., 2015) and has become an important topic of interest regarding heritage tourism destinations and tourist behavior (Zhou et al., 2015).

Revisit intention (RI), which is an important variable to measure a tourist's intention to revisit or return to a destination, is not only an important aspect of tourist behavior but also an essential indicator of the successful development of destinations (Baker and Crompton, 2000). For heritage destinations, understanding the determinants of tourists' willingness to revisit can provide managers with the foundation for managing heritage destinations. Studies have shown that the authenticity of heritage tourism is an essential factor that influences tourists' RI (Yi et al., 2017; Park et al., 2019). However, the relationship between authenticity and RI is not consistent. For example, Kolar and Zabkar (2010) proposed that objective authenticity (OA), constructive authenticity (CA), and existential authenticity (EA) affect RI (Kolar and Zabkar, 2010). Furthermore, other studies have suggested that OA and CA affect RI, whereas EA does not (Zhou et al., 2013). In addition, Park et al. (2019) explored the mediating effect of satisfaction on the relationship between authenticity and loyalty. The results showed that CA and EA affect loyalty via satisfaction, whereas OA does not correlate with satisfaction or loyalty. Thus, current research on how tourism authenticity affects tourists' RI remains unclear. This raises the question, how does tourism authenticity influence tourists' RI?

In the relationship between tourism authenticity and RI, tourism authenticity may be regarded as an environmental stimulus, and RI may be considered a human behavioral response. The cognitive appraisal theory (CAT) of the emotion theory explains that behavior is formed by the interaction between individuals and the environment, which is valuable for studying consumer behavior in the environment. According to the CAT, consumer behavior is produced in response to external stimuli. Specifically, consumers perform cognitive and emotional assessments following exposure to external stimuli, which eventually leads to specific behavior. Although tourism authenticity may not be the direct cause of RI, it may provide an environmental stimulus for RI. Memorable tourism experiences (MTEs), which are experiences that tourists actively remember after visiting a tourist destination (Kim et al., 2012), are cognitive evaluations of external stimuli (such as tourism authenticity). Relevant studies have shown that tourism authenticity correlates with MTEs (Kesgin et al., 2021). Moreover, cognitive evaluations of external stimuli produce emotional responses. Studies have shown that MTEs are associated with place attachment (PA) (Jorgensen and Stedman, 2006). In addition, emotional responses induce specific behaviors, and studies have demonstrated that PA correlates with RI (Yu et al., 2010).

Therefore, based on the CAT, this study introduced a cognitive (namely, MTEs) and an emotional variable (namely, PA) and explored the role of MTEs and PA in the relationship between tourism authenticity and RI. The study aimed to deepen our understanding of authenticity and its theoretical role in the formation of RI and provide practical guidance for the application of authenticity to heritage tourism sites.

## Literature Review

### Cognitive Appraisal Theory

The CAT is an important theory that explains consumers' response to external stimuli (Bougie et al., 2003; Soscia, 2007) and states that subjective evaluation stimuli include environmental events, personal concerns, historical experiences, and other sensitivities. Evaluation is an individual's cognitive response to a stimulant. Emotion is the psychological interpretation of the individual's cognitive evaluation of a stimulant. Consumer behavior refers to the specific behavior that can induce relevant emotions after an individual evaluates a stimulant (Bagozzi et al., 1999). Therefore, according to the CAT theory, an individual's subjective interpretation of a stimulant affects his or her cognitive evaluation and emotional response (Lazarus and Lazarus, 1991). Emotions affect behavioral responses and are an individual's adaptive meaning analysis or evaluation of the environment in regard to his or her interests (Lazarus and Lazarus, 1991). Thus, when an environment stimulates people, people will also respond to the stimulus. This form of individual evaluation of the environment involves both internal and external evaluation. Internal evaluation is the internal perceptual evaluation of personalities, beliefs, and goals, that is, the perceptual evaluation of the self. In contrast, external evaluation refers to the external perceptual evaluation of product performance and feedback from others, that is, the perceptual evaluation of the environment (Lazarus and Lazarus, 1991).

Drawing on the CAT theory, this paper explores the impact of heritage tourism authenticity on tourists' RI. Specifically, we investigated the authenticity of heritage tourism destinations as stimulus factors: MTEs as cognitive evaluation, PA as an emotion, and RI as a behavioral response.

### Authenticity in Heritage Tourism

With the development of the experience economy, tourists' demand for cultural tourism has grown (Xu et al., 2014). Heritage tourism refers to a form of tourism aimed at learning about and experiencing local culture and heritage (Poria et al., 2003). Therefore, heritage tourism is an important part of cultural tourism (Seyfi et al., 2019). Because of its association with RI, tourists' experience is extremely important for the development of destinations (Pearce, 2009). In heritage tourism, discussions around the tourist experience are usually related to authenticity, whereby the experience or product is original and authentic (Yeoman et al., 2007). As a new approach to conducting business and promoting activities, information and communication technology is currently used widely in tourism and has a significant impact on the tourist experience (Cantoni, 2020; Stylos et al., 2021). Studies have indicated that the use of social media in museums and cultural heritage can improve tourists' positive experience (Vassiliadis and Belenioti, 2017) and brand equity (Belenioti et al., 2019). In addition, virtual reality has been recognized as a powerful tool to enhance the heritage experience and is considered a supplement to the real travel experience (Mura et al., 2017).

Authenticity is a dynamic concept. OA has been described by MacCannell (1973) and Boorstin (1992), CA has been described by Cohen (1988); Bruner (1994), and EA has been described by Wang (1999). Among these, OA is considered the primitiveness of the museum style measured using objective standards (MacCannell, 1973); CA assumes that objective elements are socially constructed (i.e., a form of symbolic authenticity); and EA considers authenticity as an existential state of being (Wang, 1999). Both OA and CA are related to tourism objects, whereas EA is related to tourism subjects (Wang, 1999).

Object-based authenticity (OBA) refers to authenticity from the perspective of tourism objects. Among these, OA is the cognition of original authentic objects, which is a pure “black and white” concept of “authenticity.” Boorstin (1992) regards authenticity as an inherent attribute of tourism objects and that there is an absolute standard for measuring the authenticity of tourism objects. MacCannell (1973) and Boorstin (1992) believe that OA is genuine and authentic and emphasize the equivalence of the tourism object with the original. With the development of tourism, tourism objects gradually include traces of commercialization. However, tourists can still sense the authenticity of commercialized tourism objects. Tourism object authenticity can be understood from the perspective of social construction; thus, CA has entered people's awareness. Cohen (1988) pointed out that authenticity can be constructed and negotiated. CA is the reality created by tourism operators or authorities and is not the same as OA (Bruner, 1994). The evaluation of object authenticity is variable, and authenticity can offer true symbolic meaning. Regardless of whether authenticity is the OA of the original or the variability of CA, both emphasize the authenticity of tourism objects. With the development of the tourism experience, researchers have begun to shift their attention from tourism objects to tourism subjects, which has led to the topic of EA.

EA was proposed by Wang, who believes that people who live in modern societies and fast-paced working environments can lose their true selves easily (Wang, 1999), and only in unfamiliar territories can people find their true selves (Wang, 1999). Tourism is a particular activity in which people engage in an unfamiliar environment; therefore, tourism activities make it easy for people to find their true selves. EA, which is people-centered, describes the authenticity of personal experiences but does not destroy personal values (Kim and Jamal, 2007). Moreover, EA emphasizes the true self; according to Heidegger (1996), an individual's authentic self is the embodiment of EA. Being an authentic individual means going beyond the existing daily life state, such as one's behavior, activity, and thought, and is a state of individualistic existence.

In conclusion, tourism authenticity comprises OBA and EA. In heritage tourism, the authenticity of tourism attractions is an important factor in tourism motivation. The authenticity of cultural evolution and social construction cannot be ignored, and the authenticity of the tourism subject is indispensable. Therefore, we analyzed the authenticity of heritage tourism according to OBA and EA.

### Research Hypothesis

#### Heritage Tourism Authenticity and Revisit Intention

RI has been extensively explored as an important indicator of tourists' behavior intention and loyalty. RI refers to the possibility of revisiting destinations (Baker and Crompton, 2000). Encouraging tourists to revisit a destination is very important for ensuring the sustainable development of tourism destinations (Ali et al., 2016). Therefore, previous studies have explored the antecedents of RI to understand why tourists revisit destinations (Meleddu et al., 2015). The results have shown that these antecedents of RI include destination image and attributes (Niininen et al., 2004; Hernández-Lobato et al., 2006) and tourists' experiences, such as tourist satisfaction (Antón et al., 2017). From the perspective of authenticity, studies have shown that authenticity is related to RI. Meleddu et al. (2015) reported that tourists' subjective perception of tourism objects affects RI to some degree. In addition, Poria et al. (2003) found that perceived authenticity affects RI in heritage tourism. Furthermore, Shen et al. (2014) revealed that EA correlates with tourists' RI to cultural heritage sites. Kolar and Zabkar (2010) also suggest that OBA and EA affect RI. Moreover, specific to heritage tourism, OBA and EA affect RI. Thus, we hypothesized the following:

H1a: OBA is positively correlated with RI.

H1b: EA is positively correlated with RI.

#### Heritage Tourism Authenticity and Memorable Tourism Experiences

MTEs are tourists' positive memories and recollections of events after engaging in a tourism activity (Kim et al., 2012). Compared with other types of experiences, MTEs emphasize the memory of the experience, which promotes behavioral intention (Coudounaris and Sthapit, 2017). Currently, research has focused on the dimensions and the antecedent and outcome variables of MTEs. In terms of MTE dimensions, researchers agree that MTEs comprise multiple dimensions. Tung and Ritchie (2011) believed that MTEs are composed of four dimensions: affection, expectations, consequentiality, and recollection, whereas Kim et al. (2012) suggested that MTEs include seven dimensions, which include hedonism, novelty, etc. In regard to destination attributes, MTEs have been suggested to include seven dimensions, which include local culture, infrastructure, natural features, etc. (Kim, 2014), whereas de Freitas Coelho and de Sevilha Gosling (2018) proposed 12 dimensions of MTEs, which include environment, culture, relationships with companions, etc. Although there is variation in the division of the dimensions of MTEs, there is agreement regarding what constitutes an unforgettable tourism experience (de Freitas Coelho and de Sevilha Gosling, 2018). Antecedent variables that affect MTEs include tourists' psychological factors, such as perceived similarity (Wei et al., 2021), and destination level factors, such as destination attributes or services (Kim, 2014). Outcome variables of MTEs include loyalty, RI (Coudounaris and Sthapit, 2017; Chen and Rahman, 2018; Wong and Lai, 2021), subjective well-being (Sthapit and Coudounaris, 2018), and PA (Vada et al., 2019). Taken together, it is evident that MTEs are not only a research hotspot of experiential marketing but also an important concept for understanding and predicting consumer behavior. Therefore, MTEs are an important factor in managing consumer experience and engagement (Taheri et al., 2017).

Authenticity is central to the heritage tourism experience (Hargrove, 2002; Zatori et al., 2018) verified the relationship among participation, authenticity, and the tourist experience and found that authenticity correlates with the tourism experience. Kesgin et al. (2021) confirmed that OBA positively affects MTEs at historical sites, whereas Pine et al. (1999) suggested that people immersed in tourism activities are more likely to have unforgettable experiences. EA comes from engaging in tourism activities (Wang and Mattila, 2015); thus, tourists' participation in activities can create unforgettable experiences (Kim, 2014). Moreover, Cao et al. (2019) reported that social relationships improve tourists' memory of travel experiences in the restaurant environment. Yi et al. (2021a,b) also confirmed that EA affects unforgettable experiences in heritage tourism. In addition, research has indicated that tourism based on authentic objects and sincere interactions leads to positive memorable experiences (Domínguez-Quintero et al., 2020). Therefore, authenticity is associated with MTEs. Thus, we hypothesized the following:

Hypothesis H2a: OBA is positively correlated with MTEs.

Hypothesis H2b: EA is positively correlated with MTEs.

#### Heritage Tourism Authenticity and Place Attachment

PA refers to an individual's emotional connection with a specific environment (Eisenhauer et al., 2000), the emotional investment in a place (Hummon, 1992), or the degree of evaluation and identification with a particular environment (Moore and Graefe, 1994). PA consists of place dependence and place identity (Bricker and Kerstetter, 2000), which refer to an individual's functional (Gu and Ryan, 2008) and emotional attachment to a place, respectively (Moore and Graefe, 1994). That is, an individual or community uses places as a medium to define oneself and feel a part of the place emotionally. Currently, there is no consensus on the relationship between place identity and place dependence. Several studies have suggested that the two are independent (Kyle et al., 2005), whereas other studies have suggested that the two influence each other (Jorgensen and Stedman, 2001; Lewicka, 2011). As a complex emotion, PA is a positive result of the people–place interaction. PA affects tourist satisfaction and loyalty. Therefore, PA plays a vital role in destination management.

PA reflects the positive state of an individual when approaching a particular place. According to the CAT theory, individuals evaluate the relevance and suitability of the environment to provide personal meaning and subsequently generate emotions. In the tourism context, tourists may form an attachment to a destination because of their satisfaction, specific personal goals, or symbolic meaning.

In other words, tourists may feel a strong sense of authenticity or identify tourist destinations to meet their needs (Meng and Choi, 2016), and the satisfaction of authenticity may result in tourists' PA (Belhassen et al., 2008). Given that authenticity emphasizes the sense of place that tourists experience, when heritage tourism destinations have high OBA, tourists make a positive evaluation of the suitability of the destinations to meet their needs based on the object standard, which results in tourists' functional dependence. Moreover, tourists can obtain an even richer tourist experience through natural tourism objects, which helps promote the identity of heritage sites. In addition, when tourists participate in tourism activities at heritage sites to obtain EA, they become dependent on the heritage site because they escape from their habitual environment. Furthermore, the authentically expressed self-state is conducive to tourists' immersion and self-realization, which strengthens tourists' place identity. Indeed, studies have shown that authenticity is highly correlated with PA (Jiang et al., 2017; Park et al., 2019). Therefore, we proposed the following hypotheses:

H3a: OBA is positively correlated with PA.

H3b: EA is positively correlated with PA.

#### Memorable Tourism Experiences and Place Attachment

When engaging in tourism activities, positive experiences gained by tourists help them to become immersed in the environment, obtain higher satisfaction, and provide spiritual meaning to a specific place, which facilitates the formation of their sense of identity and dependence on a place (Tsai, 2016). In other words, positive tourist experiences determine tourists' PA to the destination (Io and Wan, 2018). MTEs are constructed by tourists according to their experience evaluations, which are used to consolidate and strengthen the pleasant memory of the destination experience; therefore, MTEs belong to the positive tourism experience (Kim, 2014), and MTEs positively affect PA (Jorgensen and Stedman, 2006). We hypothesized the following:

H4: MTEs are positively correlated with PA.

#### Memorable Tourism Experiences and Revisit Intention

Previous studies have shown that tourism experiences affect tourists' RI (Gomez-Jacinto et al., 1999); that is, past tourism experience is considered an important factor in determining whether destinations are revisited (Chandralal and Valenzuela, 2013). RI is deemed a positive outcome of MTEs (Tung and Ritchie, 2011; Kim et al., 2012; Marschall, 2012). Research has demonstrated that there is a correlation between MTEs and RI (Kim et al., 2012), and MTEs are an essential factor in predicting RI (Kim, 2014). Chen and Rahman (2018) found that MTEs of cultural tourism affect loyalty. In addition, Rasoolimanesh et al. (2021) proposed that memorable heritage tourism experiences influence whether tourists return and their recommendations. Therefore, we offered the following research hypothesis:

H5: MTEs are positively correlated with RI.

#### Place Attachment and Revisit Intention

In tourism experiences, the intensity of PA determines tourists' willingness to revisit the destination. George and George (2004) considered PA as an important antecedent of RI. However, there are differences in the influence of place dependence and place identity on RI (Prayag and Ryan, 2012; Scarpi et al., 2019). Moreover, research has suggested that the PA of tourists to heritage tourism destinations affects RI (Ding et al., 2015). Thus, we proposed the following hypothesis:

H6: PA is positively correlated with RI.

**Figure 1:**
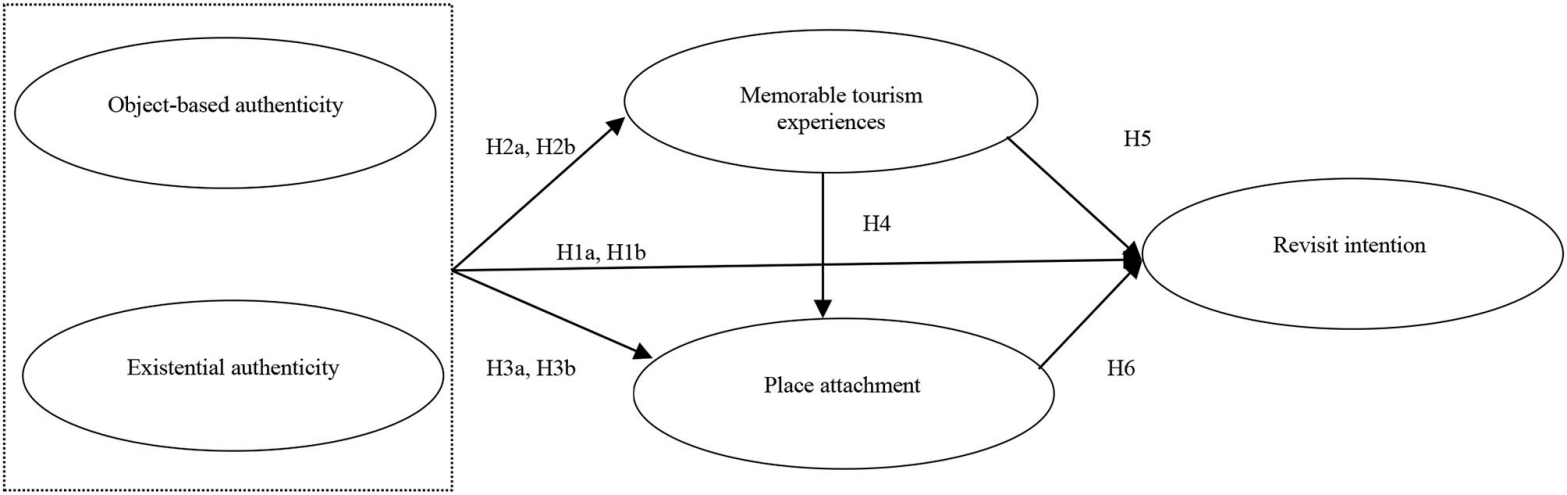
Conceptual model.

The concept model is shown in Figure 1.

## Methodology

### Research Site

The Dujiangyan irrigation system, which is one of the world heritage sites of China, served as our study context. The Dujiangyan irrigation system is an ecological engineering feat located in Dujiangyan City in Sichuan Province. It is the world's largest water conservancy project and includes Yuzui, Feisha weir, and Baopingkou. The Dujiangyan irrigation system has beautiful scenery and numerous cultural relics, such as the Fulong Temple, Erwang Temple, Anlan Cable Bridge, Yulei Pass, Lidui Park, Yulei Mountain Park, and the resulting hydrology of water, God, and human sacrifices. In line with UNESCO's statement, the Dujiangyan irrigation system has a history of more than 2,000 years since its establishment and is still in use today, and internationally recognized protection guidelines and rules have been followed to protect and restore projects. The Dujiangyan irrigation system was largely undamaged by the Wenchuan earthquake on May 12, 2008 (https://whc.unesco.org/en/list/1001). In addition, to celebrate the completion of the Dujiangyan irrigation system, the beginning of the busy spring farming and production season, and the commemoration of Li Bing, a large-scale celebration called the Water Release Festival is held, during the Tomb-Sweeping Festival of the 24 solar terms of the lunar calendar that occurs every year in China, which reproduces the grand occasion of the Dujiangyan water release more than 2,000 years ago. The Dujiangyan Water Release Festival is now part of China's national cultural heritage.

### Variable Measurement

We used established scales from relevant literature to measure the variables, and all items were adjusted appropriately in heritage tourism context. Authenticity was measured using the scales developed by Kolar and Zabkar (2010). OBA refers to the authenticity of object orientation and includes four items (e.g., “During my visit to the Dujiangyan irrigation system, I perceived that the overall layout or environment is original”). EA reflects the status of activity orientation and includes six items (e.g., “During my visit to the Dujiangyan irrigation system, I was freed from daily routines and became more of myself”). From the study of Kim et al. (2012) and Lee (2015), we adopted six items (e.g., “I enjoyed the experience and felt excited”) to measure MTEs, which reflect tourists' evaluation of the tourism experience. From the study of Prayag and Ryan (2012), we adopted six items (e.g., “This destination is very special to me”) to measure PA, which reflect tourists' emotional attachment to a particular destination. RI reflects loyalty to the destination and included four items proposed by Backman and Crompton (1991) and Morais and Lin (2010) (e.g., “I would like to visit the Dujiangyan irrigation system again”). A seven-point Likert scale (from “strongly disagree” to “strongly agree”) was used for all constructs.

### Data Collection and Sample

Self-report is an easy and effective data collection method (Koslowsky and Dishon-Berkovits, 2001), which is the most used in the study of tourist behavior. Relevant studies believed that the impact of self-report on common method variation (CMV) is limited (Fox and Spector, 1999). Therefore, we collected data using a self-reported questionnaire. The survey lasted for 2 months from July 2021 to August 2021, during the Dujiangyan irrigation system's peak tourist season. A total of 620 questionnaires were completed, and 596 valid questionnaires were collected. Among the 596 observations, 54% were female; 42% and 40% were aged 21–34 years and 35–50 years, respectively, and 85% had a bachelor's degree or higher. In terms of occupation distribution, 46% were company employees, and 17, 13, and 14% were self-employed, working at government institutions, and working at public institutions, respectively. For income, 70% earned more than 5,000 RMB per month.

## Results

### Measurement Model

We analyzed 596 valid samples using AMOS 22.0. The mean value of each item ranged from 4.85 to 5.82. CFA was conducted to evaluate whether data were consistent with the measurement model. The results showed that χ^2^/DF = 2.858, RMSEA = 0.056, SRMR = 0.0421, CFI = 0.923, IFI = 0.923, and TLI = 0.913. Reliability analysis included Cronbach's alpha and composite reliability (CR). As shown in Table 1, Cronbach's alpha (0.705–0.859) and CR (0.8207–0.8954) exceed the recommended standard of 0.70, which indicated good reliability. Standard factor loadings (0.629–0.819) were higher than 0.6 and significant at *p* < 0.001, as shown in Table 1. Average variance extractions (AVEs, 0.5349–0.6069) were >0.5. The square root of the AVE was higher than the correlation coefficient between corresponding latent constructs (presented in Table 2), which indicated good discriminant validity.

**Table 1:** Reliability coefficients and the average variance extractions (AVEs) of the variables.

**Variables**	**Items**	**Standardization factor load**	**S.E**.	***T*** **-value**	**Cronbach's α**	**CR**	**AVE**
Object-based authenticity	OBA 1	0.778			0.783	0.8606	0.6069
	OBA 2	0.772	0.080	13.069			
	OBA 3	0.774	0.081	13.137			
	OBA 4	0.792	0.086	13.466			
Existential authenticity	EA 1	0.769			0.838	0.8831	0.559
	EA 2	0.819	0.068	17.122			
	EA 3	0.709	0.073	14.008			
	EA 4	0.785	0.070	16.260			
	EA 5	0.760	0.067	15.153			
	EA 6	0.629	0.070	11.904			
Memorable tourism experiences	MTEs 1	0.727			0.828	0.8748	0.5381
	MTEs 2	0.725	0.079	13.188			
	MTEs 3	0.726	0.078	13.212			
	MTEs 4	0.749	0.083	13.703			
	MTEs 5	0.742	0.079	15.558			
	MTEs 6	0.732	0.087	13.405			
Place attachment	PA 1	0.789			0.859	0.8954	0.5883
	PA 2	0.733	0.064	15.433			
	PA 3	0.781	0.062	16.867			
	PA 4	0.807	0.061	17.686			
	PA 5	0.745	0.057	15.775			
	PA 6	0.744	0.058	15.718			
Revisit intention	RI 1	0.796			0.705	0.8207	0.5349
	RI 2	0.761	0.086	11.113			
	RI 3	0.660	0.080	9.685			
	RI 4	0.701	0.080	10.535			

**Table 2:** Discriminant validity of the variables.

**Variable**	**1**	**2**	**3**	**4**	**5**
1. OBA	0.7790				
2. EA	0.685**	0.7477			
3. MTEs	0.682**	0.697**	0.7334		
4. PA	0.649**	0.661**	0.678**	0.7670	
5. RI	0.560**	0.574**	0.513**	0.572**	0.7314
Mean value	5.2969	5.6755	5.2292	5.3079	5.4223
Standard deviation	0.83558	0.78646	0.84315	0.85860	0.83415

** *Significantly correlated at p < 0.01 level (bilateral) and the square root of AVE was on the diagonal*.

### Descriptive Statistical Analysis

As shown in Table 2, OBA was significantly positively correlated with MTEs (*r* = 0.682, *p* < 0.01), PA (0.649), and RI (0.560). EA was significantly positively correlated with MTEs (0.697), PA (0.661), and RI (0.574). In addition, MTEs were significantly positively correlated with PA (0.678) and RI (0.513). Finally, there was also a significant positive correlation between PA and RI (0.572).

### Structural Model

AMOS 22.0 was used to test research hypothesis, and the results are presented in Table 3.

**Table 3:** Structural equation modeling results.

**Hypothesized path**	**Standardized coefficients**	**Standard error**	**T-value**	**Hypothesis**
H1a OBA—RI	0.371*	0.144	2.443	Supported
H1b EA—RI	0.284*	0.154	2.290	Supported
H2a OBA—MTEs	0.529***	0.101	5.193	Supported
H2b EA—MTEs	0.388***	0.128	3.919	Supported
H3a OBA—PA	0.283*	0.144	2.442	Supported
H3b EA—PA	0.202*	0.125	2.067	Supported
H4 MTEs—PA	0.392***	0.102	3.824	Supported
H5 MTEs—RI	−0.184	0.130	−1.365	Not supported
H6 PA—RI	0.360***	0.090	3.891	Supported

* *p < 0.05*,

*** *p < 0.001*.

The SEM results verified our proposed model. Authenticity significantly positively affected RI (OBA was β = 0.371, *p* < 0.05; EA was β = 0.284, *p* < 0.05). Therefore, H1a and H1b were supported.

Additionally, both OBA and EA significantly positively affected MTEs (OBA was β = 0.529, *p* < 0.001; EA was β = 0.388, *p* < 0.001). Therefore, H2a and H2b were supported. In addition, both OBA and EA significantly positively affected PA (OBA was β = 0.283, *p* < 0.05; EA was β = 0.202, *p* < 0.05). Therefore, H3a and H3b were supported.

MTEs significantly positively affected PA (β = 0.392, *p* < 0.001) but not RI (β = −0.184*, p* > 0.05). As such, H4 was supported, whereas H5 was not supported. PA significantly positively affected RI (β = 0.360*, p* < 0.001). Thus, H6 was also supported.

The direct, indirect, and total effects of the constructs under analysis are presented in Table 4. OBA had the largest impact on RI, followed by EA. Regarding cognition, MTEs indirectly affected RI. As for emotion, PA indirectly impacted the relationship between authenticity and RI and between MTEs and RI.

**Table 4:** Direct, indirect, and total effects of relationships.

**Path**	**Direct**	**Indirect**	**Total**
H1a OBA—RI	0.371*	0.079*	0.450*
H1b EA—RI	0.284*	0.056*	0.340*
H2a OBA—MTEs	0.529***		0.529***
H2b EA—MTEs	0.388***		0.388***
H3a OBA—PA	0.283*	0.207*	0.490*
H3b EA—PA	0.202*	0.152*	0.354*
H4 MTEs—PA	0.392***		0.392***
H5 MTEs—RI	−0.184	0.141*	−0.043
H6 PA—RI	0.360***		0.360***

* *p < 0.05*,

** *p < 0.01, and*

*** *p < 0.001*.

MTEs and PA played a mediating role in the relationship between authenticity and RI. We used bootstrap analysis to test the mediating role of MTEs and PA (Preacher and Hayes, 2004; Hayes, 2013). The results showed that the confidence intervals (CIs) for MTEs on the relationships between OBA and RI (lower limit [LL] = 0.0993, upper limit [UL] = 0.2350) and between EA and RI (LL = 0.0941, UL = 0.2305) did not include zero, and the size of mediation effect ranged from 0.1675 to 0.1634 (Table 5). The CIs for PA on the relationships between OBA and RI (LL = 0.1662, UL = 0.3134) and between EA and RI (LL = 0.1731, UL = 0.3201) did not include zero, and the size of mediating effect ranged from 0.2332 to 0.2398. Similarly, the CIs of MTEs and PA on the relationships between OBA and RI (LL = 0.1964, UL = 0.3634) and between EA and RI (LL = 0.2118, UL = 0.3631) did not include zero, and the size of mediating effect ranged from 0.2776 to 0.2826.

**Table 5:** Outputs of the bootstrap test of the mediating effects.

**Indirect effect**	**Effect (SE)**	**LL 95% CI**	**UL 95% CI**
OBA-MTEs-RI	0.1675 (0.0348)	0.0993	0.2350
EA-MTEs-RI	0.1634 (0.0346)	0.0941	0.2305
OBA-PA-RI	0.2332 (0.0379)	0.1662	0.3134
EA-PA-RI	0.2398 (0.0367)	0.1731	0.3201
OA-MTEs-PA-RI	0.2776 (0.0424)	0.1964	0.3634
OBA-MTEs-PA-RI	0.2826 (0.0386)	0.2118	0.3616

*LL, lower limit; CI, confidence interval; UL, upper limit; SE, standard error; unstandardized regression coefficients are reported; bootstrap sample size = 5,000*.

## Discussion

The authenticity of heritage tourism destinations is an important factor for attracting tourists. Previous studies have shown that authenticity is related to RI. Therefore, we explored the impact of authenticity on RI in the context of the CAT theory and reached the following conclusions: (1) authenticity affects MTEs, PA, and RI; (2) MTEs affect PA but have no effect on RI; (3) PA affects RI; (4) both MTEs and PA mediate the relationship between authenticity and RI.

First, tourism authenticity affected RI, which is identical with the conclusion in the past (Kolar and Zabkar, 2010; Yi et al., 2017). Tourists were more likely to revisit destinations if they perceived heritage destinations as authentic. Specifically, when tourists perceived a high level of OBA, they were likely to revisit the destination. Moreover, tourists were more likely to revisit the destination when they perceived a high level of EA from relevant activities provided by the heritage tourism destination.

Second, authenticity affected MTEs, which is consistent with the findings of Domínguez-Quintero et al. (2020). This may be because authenticity is related to the tourism experience (Zatori et al., 2018). Specifically, when tourists perceive a high level of authenticity, they will make positive comments on the tourism experience, which eventually leads to high memorability. Therefore, managers must design and provide an authentic experience to meet tourists' expectations and needs and ensure that tourists obtain MTEs. In addition, authenticity affected PA, which is identical with the conclusion in the past (Jiang et al., 2017; Park et al., 2019). In tourism experiences, authenticity emphasizes the sense of place during a tourism experience. Specifically, when heritage tourism destinations provide a high level of OBA and EA, tourists achieve functional and emotional satisfaction, which promotes the formation of PA.

Furthermore, the current study confirmed a significant positive effect of MTEs on PA, which is consistent with the findings of Jorgensen and Stedman (2006) and Vada et al. (2019). MTEs are a vital antecedent variable for PA. When tourists perceive that the heritage tourism destination meets their authenticity needs, they regard destinations as memorable because they experience the original object, obtain the true self, and form a PA to the heritage destination. Moreover, they become more willing to revisit the destination.

### Theoretical Implications

This study extended the existing model of authenticity and RI by applying the CAT theory. Previous studies have shown that authenticity affects RI (Kolar and Zabkar, 2010; Zhou et al., 2013), which led subsequent studies to examine the relationships among authenticity, satisfaction or perceived value, and RI (Park et al., 2019; Su et al., 2021). However, these models focused on the cognitive variables and ignored the psychological process of tourists' reactions to authenticity. Research has suggested that cognitive and emotional variables play an important role in consumer behavior (Song and Qu, 2017). Based on the CAT theory, our study introduced cognitive (i.e., MTEs) and affective variables (i.e., PA) and hypothesized that environmental stimulation (i.e., authenticity) of heritage tourism destinations affects tourists' cognitive evaluation (i.e., MTEs), which subsequently affects tourists' behavior (i.e., RI) via an emotional response (i.e., PA). Because this theoretical model focuses on tourists' cognitive and emotional responses to environmental stimuli, it provides a deeper understanding of the relationship between authenticity and RI.

This study revealed a potentially important, yet previously unexamined, mechanism of the relationship between authenticity and RI. Previous studies have proposed that MTEs play a mediating role in the relationship between authenticity and RI (Rasoolimanesh et al., 2021). Other studies have suggested that authenticity affects RI via PA (Shang et al., 2020; Yi et al., 2021b). Drawing on the CAT theory, we found that both MTEs and PA play an independent mediating role successively between authenticity and RI, which is consistent with previous studies (Shang et al., 2020; Rasoolimanesh et al., 2021; Yi et al., 2021a). In addition, MTEs and PA played a multiple-step mediating role in the relationship between authenticity and RI. This mechanism depicts tourists' psychological process underlying the relationship between authenticity and RI.

Finally, our findings enrich the research on the relationship between MTEs and PA. We examined the relationship between MTEs and PA to heritage tourism destinations and further verified the findings of Jorgensen and Stedman (2006). Previous studies explored the relationship between MTEs and PA by including MTEs as an independent variable and PA as a dependent variable (Tsai, 2016; Vada et al., 2019). However, few studies have considered MTEs and PA as multiple mediating variables. Thus, our examination of the multiple mediating effects of MTEs and PA in heritage tourism enriches the research on the relationship between MTEs and PA.

### Practical Implications

This study focused on tourists' behavior in regard to the authenticity of heritage tourism to provide practical guidance for improving heritage tourism destinations. The local authorities of heritage tourism destinations should maintain the authenticity of both tangible (e.g., the architecture) and intangible (e.g., local legends and stories) elements and offer activities that trigger a sense of authenticity in tourists to encourage revisits. Specifically, local authority marketers and managers should strive to protect and understand OBA and design tourism activities that drive EA. This requires maintaining the authenticity of local buildings and legends and considering projects that tourists are likely to participate in when designing authentic tourism activities. Furthermore, tourism activities should help introduce tourists to local culture and enable them to communicate with residents in a natural, sincere, and friendly manner.

Heritage tourism destination managers should also aim to promote MTEs and PA by facilitating OBA and EA. This requires local managers to promote the organic unity of authenticity protection and utilization. Therefore, heritage tourism destinations should strive to unify economic and social benefits to protect and utilize authenticity. The tourist experience should be optimized by coordinating between OBA and EA to generate MTEs and form PA.

Our study revealed that MTEs significantly increase PA. This requires managers of heritage tourism destinations to recognize the importance of MTEs and carefully design products and services based on authenticity, create MTEs for tourists, and improve tourists' PA to heritage tourism destinations.

Our research also shows that PA significantly increases RI. PA is an individual's evaluation and recognition of a specific environment (Moore and Graefe, 1994). Tourists' attachment to a tourist destination requires positive interactions, where the stronger the attachment to a place, the more the tourists' willingness can be stimulated to revisit the tourist destination. Therefore, managers of heritage tourism destinations should take into account that during a tourism experience, tourists form attachments to destinations, which encourages them to revisit.

### Limitations

Although this paper focuses on the role of MTEs and PA in the influence of heritage tourism authenticity on revisit intention, this relationship is not unique, and follow-up research should also consider cognitive variables, such as destination image and perceived value, and emotional variables, such as nostalgia. The survey only acquired cross-sectional data over 2 months. To improve the robustness and effectiveness of the findings, the survey should be conducted during different periods in future.

## Data Availability Statement

The raw data supporting the conclusions of this article will be made available by the authors, without undue reservation.

## Author Contributions

GZ and WC conceived the study. GZ, WC, and YW wrote the manuscript. All authors designed the study, collected, analyzed the data, read, approved the manuscript, and agreed to be accountable for all aspects of the work.

## Funding

This research was funded by the Sichuan Centre for Rural Development Research (Grant No. CR2011) and the Special Program of Social Sciences of Sichuan Agricultural University 2019.

## Conflict of Interest

The authors declare that the research was conducted in the absence of any commercial or financial relationships that could be construed as a potential conflict of interest.

## Publisher's Note

All claims expressed in this article are solely those of the authors and do not necessarily represent those of their affiliated organizations, or those of the publisher, the editors and the reviewers. Any product that may be evaluated in this article, or claim that may be made by its manufacturer, is not guaranteed or endorsed by the publisher.
